# Characterization and density control of GaN nanodots on Si (111) by droplet epitaxy using plasma-assisted molecular beam epitaxy

**DOI:** 10.1186/1556-276X-9-682

**Published:** 2014-12-17

**Authors:** Ing-Song Yu, Chun-Pu Chang, Chung-Pei Yang, Chun-Ting Lin, Yuan-Ron Ma, Chun-Chi Chen

**Affiliations:** 1 Department of Materials Science and Engineering, National Dong Hwa University, Hualien 97401, Taiwan; 2 Institute of Photonic System, National Chiao Tung University, Tainan 71150, Taiwan; 3 Department of Physics, National Dong Hwa University, Hualien 97401, Taiwan; 4 National Nano Device Laboratories, Hsinchu 30078, Taiwan

**Keywords:** Molecular beam epitaxy, Gallium nitride, Quantum dots, Scanning photoemission microscopy, Reflection high-energy electron diffraction, Droplet epitaxy, Reflection high-energy electron diffraction, X-ray photoelectron spectroscopy

## Abstract

In this report, self-organized GaN nanodots have been grown on Si (111) by droplet epitaxy method, and their density can be controlled from 1.1 × 10^10^ to 1.1 × 10^11^ cm^-2^ by various growth parameters, such as substrate temperatures for Ga droplet formation, the pre-nitridation treatment of Si substrate, the nitridation duration for GaN crystallization, and *in situ* annealing after GaN formation. Based on the characterization of *in situ* RHEED, we can observe the surface condition of Si and the formation of GaN nanodots on Si. The surface nitridaiton treatment at 600°C provides a-SiNx layer which makes higher density of GaN nanodots. Crystal GaN nanodots can be observed by the HRTEM. The surface composition of GaN nanodots can be analyzed by SPEM and μ-XPS with a synchrotron x-ray source. We can find GaN nanodots form by droplet epitaxy and then *in situ* annealing make higher-degree nitridation of GaN nanodots.

## Background

Group-III nitride based semiconductors have been successful commercialized as light emitting diodes (LED) and high electron mobility transistors (HEMT) [1,2]. An enormous interest in gallium nitride (GaN) nanostructures can be observed due to their strong carrier confinement phenomenon. GaN quantum dots (QDs) are highly potential materials for the applications in electronics such as single electron transistors, and in optoelectronics such as QD lasers, single photon source and photodetectors [3,4]. Recently, high-density GaN nanodots and nanorods are also expected to be illuminated working electrodes of photoelectrochemical water splitting to generate hydrogen gas. The hydrogen evolution by natural energy will be an important technique to prevent the global warming in the future [5].

For the fabrication of self-assembled semiconductor nanostructures, several methods were proposed so far. For instance, molecular beam epitaxy (MBE) and metal organic chemical vapor deposition (MOCVD) provided nanodots growth via Stranski-Krastanov (SK) mode, which requires sufficient lattice mismatch between substrate, wetting layer and epi-layer [6-10]. However, there was still a challenge to have higher density, to easily control the growth, and to have fewer defects of self-organized semiconductor nanostructures. In the last ten years, the droplet epitaxy (DE) technique is another method to obtain nanostructures with some advantages over the SK mode. For example, a variety of quantum structures have been obtained by this technique such as dots, rings, holes, wires, dot pairs, dot disks, which can be fabricated on any sort of substrates. This flexible nanostructure-fabrication technique can apply to a wide range of materials by precisely controlling the lateral diffusion of metallic droplets and crystallization process [11,12]. Besides, many applications have been proposed by droplet epitaxy technique: GaAs/AlGaAs quantum dot laser [13], single photon emitter on Si [14], and infrared photodetector with strain-free GaAs quantum dot pairs [15].

For the growth of GaN nanodots by droplet epitaxy technique, Ga droplets formation and then nitridation process for GaN crystallization, has been employed to fabricate GaN nanodots on c-plane sapphire, Si (111), 6H-SiC (0001) and Si_3_N_4_(0001)/Si(111). The size and density of GaN nanodots were investigated by different Ga flux and substrate temperatures from the results of atomic force microscopy (AFM) [16-18]. For the first part of droplet epitaxy: Ga droplet formation, its growth mechanism has been experimentally and theoretically investigated in terms of nucleation, coalescence and ripening processes. Droplet number density can be easily controlled by a suitable choice of substrate temperature [19-21]. However, for the second part of droplet epitaxy: GaN crystallization, GaN nanodot formation was not a simple transformation of Ga droplets into GaN dots. Additional phenomena like surface diffusion of Ga and formation of a rough layer may play a role. Therefore, some further investigation is necessary to understand the growth mechanism of GaN nanodots by droplet epitaxy [22].

In this report, GaN nanodots were grown on Si (111) substrate by droplet epitaxy using plasma-assisted MBE system at various growth parameters. We focused on the characterization of GaN nanodots by scanning photoemission microscopy (SPEM) and x-ray photoelectron spectroscopy (XPS) from a synchrotron radiation x-ray source. *In situ* reflection high-energy electron diffraction (RHEED) was employed to observe Si surfaces and GaN nanodots. *Ex situ* transmission electron microscopy (TEM) was conducted to analyze the crystalline of GaN nanodots. In order to obtain higher density of GaN nanodots on Si for future applications, we also investigated the density of GaN nanodots by controlling the substrate temperatures, nitridation time for GaN crystallization, the pre-nitridation treatment of substrates, and *in situ* annealing after GaN nanodot formation from the images of field emission scanning electron microscopy (FESEM).

## Methods

**Figure 1:**
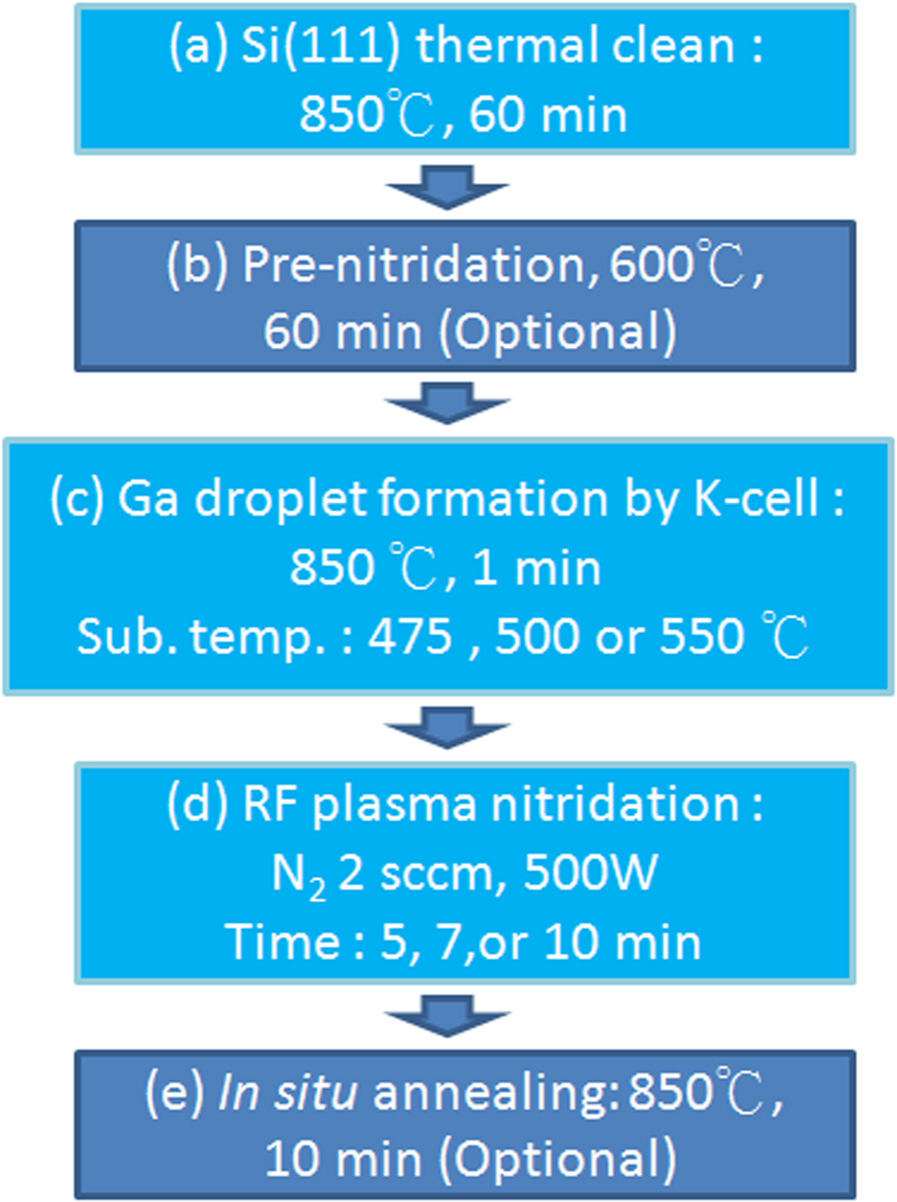
**Process flow and parameters for the growth of GaN nanodots. (a)** Thermal cleaning. **(b)** Substrate pre-nitridation treatment (optional). **(c)** Ga droplet formation. **(d)** RF plasma nitridation for GaN formation. **(e)** In situ annealing treatment (optional).

GaN nanodots by droplet epitaxy were carried out in our ULVAC MBE system with a radio frequency (RF) nitrogen plasma source. The process flow and parameters of GaN nanodots formation is shown in Figure 1. Si (111) wafers were cleaned by acetone to remove organic impurity, cleaned by 10% HF solution to remove the native oxide, and then put into MBE chamber immediately. Until the base pressure of chamber lower than 1.0 × 10^-7^ Pa, thermal cleaning of Si substrates was conducted at 850°C for 60 min. Some of the samples had pre-nitridation treatment on Si substrates at temperature 600°C for 60 min. Then, Ga droplets were deposited by Ga Knudsen cell at 850°C for 1 min (beam equivalent pressure 1.9 × 10^-4^ Pa) at substrate temperatures 475°C, 500°C and 550°C. Nitridation process was followed to form GaN nanodots for 5 min, 7 min or 10 min. For the processes of Ga droplets nitridation and substrate pre-treatment, nitrogen plasma source operated at a RF forward power of 500 W and N_2_ flux of 2 sccm, which provided beam equivalent pressure 1.2 × 10^-5^ Pa. After the formation of GaN nanodots, *in situ* annealing at 850°C for 10 min was the optional process for some samples.

During the fabrication of each sample, surface quality and structure was monitored by *in situ* RHEED with electron beam energy 20 KeV. For the characterization of GaN nanodots, SPEM and μ-XPS were conducted on beamline U5 at the National Synchrotron Radiation Research Center (NSRRC) in Hsinchu, Taiwan, which can suffer photon energies from 60 to 1200 eV during high-resolution XPS measurement and provide photon beam size 90–100 nm in diameter [23]. The crystallinity of GaN nanodots was studied by high-resolution TEM, JEOL JEM-2010 F with accelerating voltage 200 KV. Ga droplets and GaN nanodots on Si were observed by a JEOL FESEM with accelerating voltage 15 KV, and their densities were obtained from SEM images.

## Results and discussion

### In situ RHEED analysis

**Figure 2:**
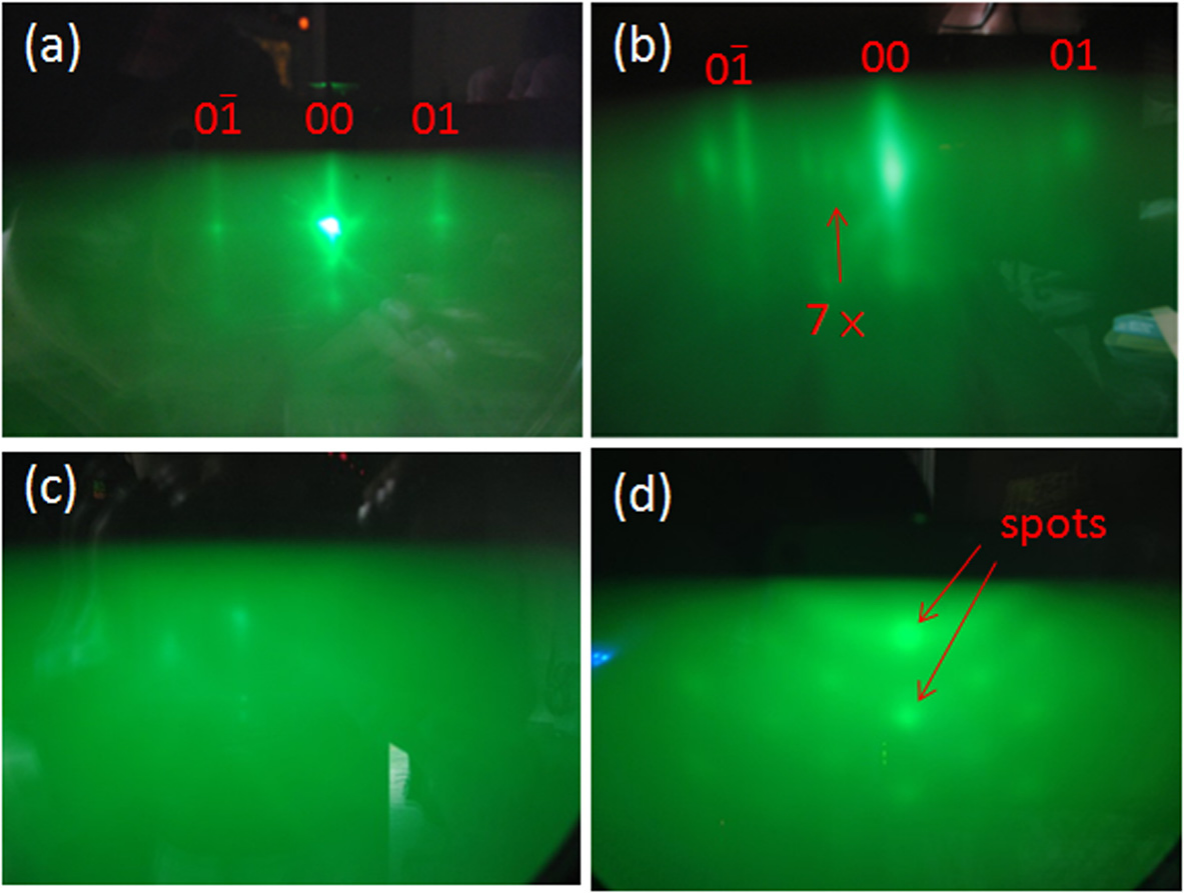
**RHEED patterns. (a)** Reflections of the Si (111) before thermal cleaning. **(b)** Si (111)-7 × 7 surface reconstruction after thermal cleaning. **(c)** Reflection of the Ga droplets on Si. **(d)** Reflection of crystal GaN nanodots on Si.

Figure 2(a) shows the *in situ* RHEED pattern of Si (111) before thermal cleaning, and a smooth surface gives long streaks in the RHEED pattern. After the thermal cleaning at 850°C for 60 min, Si (111)-7 × 7 reconstruction appears as shown in Figure 2(b). After the formation of Ga droplets on Si, RHEED pattern becomes cloudy as shown in Figure 2(c), which means a thin amorphous layer on Si. Followed by RF plasma nitridation, GaN nanodots formed on Si. Spotty and foggy RHEED pattern presents due to the rough surface by GaN nanodots on Si, shown in Figure 2(d). From the results of RHEED, we can find the pattern transition from 2D diffraction surface normal streaks to 3D diffraction Bragg spots during the growth of GaN nanodots by droplet epitaxy.

**Figure 3:**
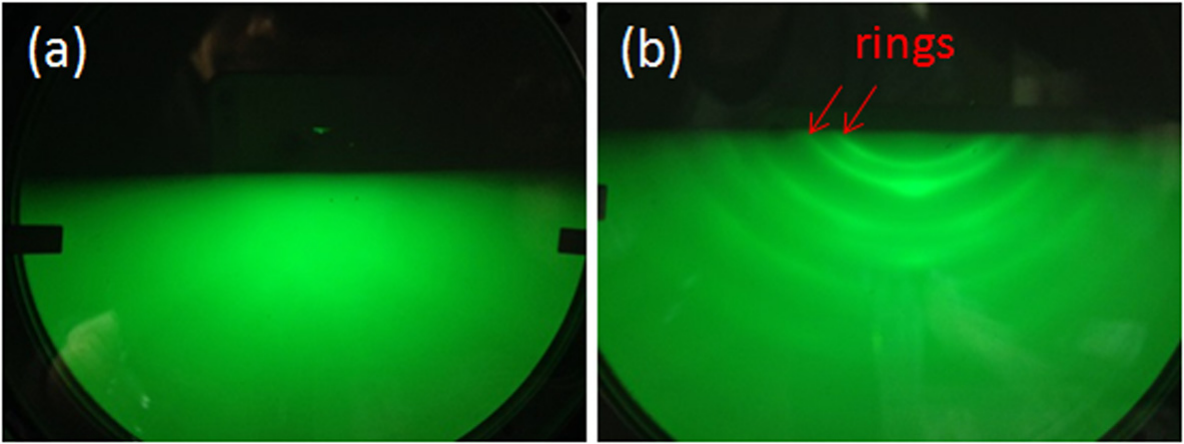
**RHEED patterns. (a)** Cloudy pattern shows an amorphous nitride layer on Si substrate after pre-nitridation treatment at 600°C for 60 min. **(b)** The rings-centered pattern indicates polycrystalline of GaN nanodots on Si.

Some of the samples were prepared with pre-nitridation treatment at 600°C for 60 min in order to create different Si surface for the growth of GaN nanostructure. After the pre-nitridation, entirely cloudy RHEED pattern is in appearance due to the amorphous nitride layer on Si. Low-temperature pre-nitridation on Si substrate can produce an amorphous nitride layer for Ga deoplet formation. This result is different from the report by Gwo’s group. Single-crystal β-Si_3_N_4_ layer was formed by nitrogen plasma nitridation at 830°C from their RHEED observation [18]. After the growth of GaN nanodots on this sample, RHEED pattern gives rings centered as shown in Figure 3(b), which comes from the polycrystalline of GaN nanodots. From the results of *in situ* RHEED, we can not only observe the growth of GaN nanodots, but also determine the surface condition which influences the growth and density of GaN nanodots (discuss latter).

### Ex situ SEM and TEM analysis

**Figure 4:**
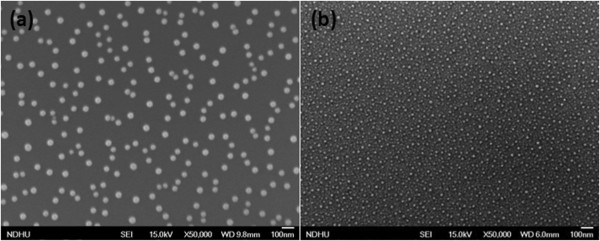
**SEM images. (a)** Ga droplets density 6.45 × 109 cm-2 and their size around 60 nm. **(b)** GaN nanodots density 5.01 × 1010 cm-2 and their size around 15 nm.

**Figure 5:**
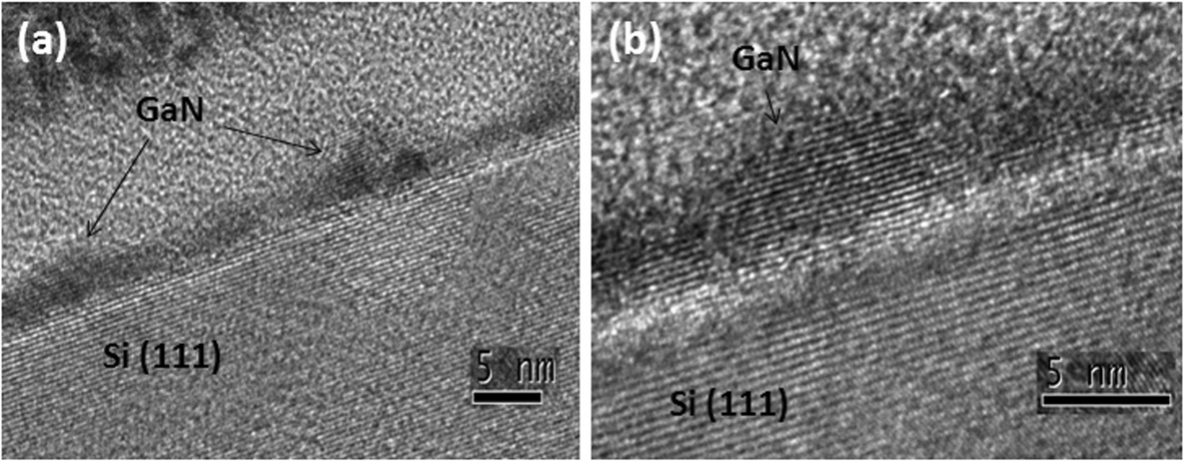
**High-resolution cross-sectional TEM images. (a)** GaN nanodots on Si (111), and **(b)** One crystal GaN nanodot on Si in higher magnification.

Figure 4(a) is the SEM image of Ga droplets on Si. This sample was grown at substrate temperature 550°C by K-cell 850°C for 1 min. The density of Ga droplets is 6.45 × 10^9^ cm^-2^, and the average size is around 60 nm. For the same condition of Ga droplet formation, another sample formed GaN nanodots by RF plasma nitridation for 10 min as shown in Figure 4(b), their density becomes 5.01 × 10^10^ cm^-2^, and the average size is around 15 nm. During the GaN crystallization, GaN nanodot formation was not a simple transformation of one Ga droplet into one GaN dot and their growth mechanism was more complex. To forward discussing their growth mechanism, the kinetics of Ga droplet formation was well studied: Ga droplets nucleate heterogeneously at the lower-energy sites on the surface or nucleate homogeneously. Then, Ga droplets grow and coalesce [24,25]. After droplet formation, sample was immediately served nitrogen source by RF plasma, Ga atoms diffused on the surface quickly and reacted with limited nitrogen source to form GaN dots. It is the reason that one Ga droplet can form several GaN nanodots as the report in reference 22.For the sample with the SEM image of Figure 4(b), high-resolution cross-sectional TEM was conducted to observe GaN nanodots. In Figure 5(a), GaN nanodots can be observed on Si, their size is around 10 nm. For the higher magnification of TEM image in the Figure 5(b), we can find the GaN nanodot crystal and a thin amorphous layer with thickness 1.5 nm between GaN nanodots and Si (111) substrate.

### SPEM and μ-XPS analysis by synchrotron x-ray

**Figure 6:**
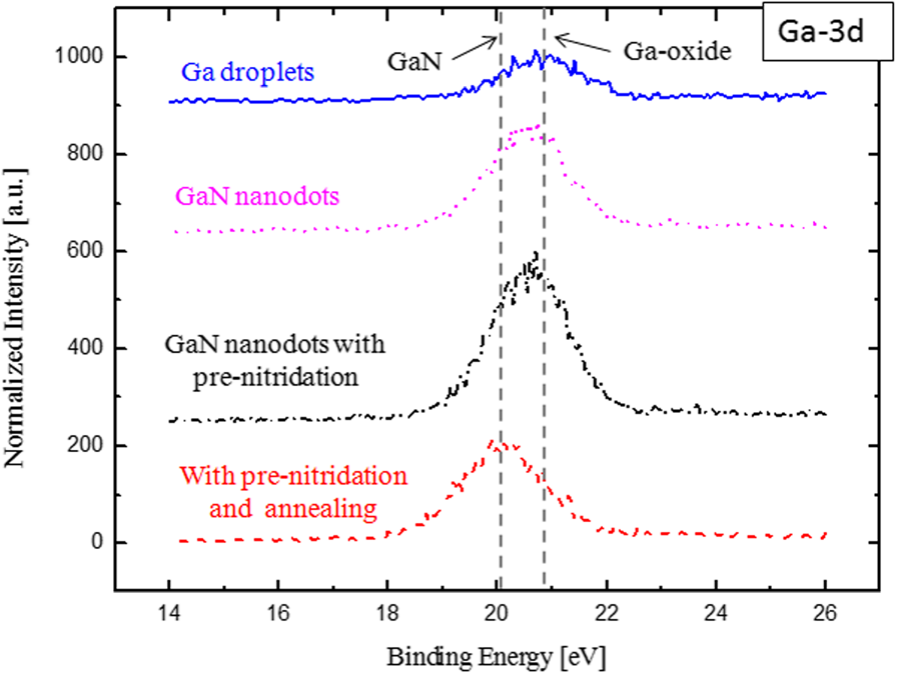
**Ga-3d XPS core-level spectra for four samples.** Ga droplets on Si (blue line), GaN nanodots on Si (pink line), GaN nanodots on Si with substrate pre-nitridation (black line), and GaN nanodots on Si with substrate pre-nitridation and in situ annealing (red line). The Ga-3d XPS peaks shift from 21 eV to 20 eV, which means the formation of GaN nanodots.

SPEM and μ-XPS were used to analyze the surface composition of GaN nanodots on Si (111). For chemical state mapping of Ga-3d with block size 5 μm × 5 μm, we got 16-channel SPEM images with binding energy from 14 eV to 26 eV. Because of the high density of GaN nanodots on Si (5.01 × 10^10^ cm^-2^, see the SEM image of Figure 4(b)), the resolution of SPEM mapping did not good enough to clearly identify single GaN dot, so the result is not shown here. To control the density of GaN nanodots, we are going to discuss in the following section. The second mode of a SPEM system is photoelectron spectroscopy from a small spot area, so-called μ-XPS. We conducted the measurements on 4 samples: Ga droplets (see SEM image of Figure 4(a)), GaN nanodots (see SEM image of Figure 4(b)), GaN nanodots with substrate pre-nitridation, GaN nanodots with pre-nitridation and annealing. Figure 6 shows Ga-3d XPS spectra for these 4 samples. The sample with Ga droplets has peak at 21 eV, which can be attributed to oxidized Ga due to exposure to air. As the GaN started to form by nitridation, the XPS peak of this sample shifts to the binding energy site of Ga-N, which corresponds to the formation of GaN. The sample with substrate pre-nitridation has similar XPS results with the one without pre-nitridation. The pre-nitridation of Si substrate did not influence the chemical composition of GaN nanodots on Si. Furthermore, the Ga-3d XPS peak shifts to 20 eV corresponding to the peak of GaN. We can find the annealing process in MBE made higher-degree nitridation of GaN nanodots.

### Density analysis of GaN nanodots

**Figure 7:**
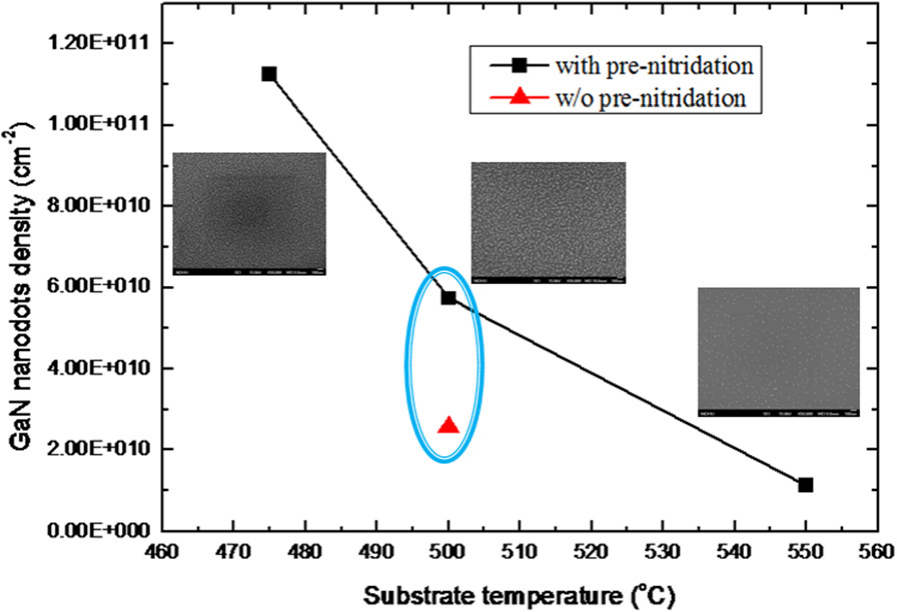
**Density of GaN nanodots as function of substrate temperatures at 475°C, 500°C and 550°C and their SEM images.** Density of GaN nanodots for two samples (in the blue cycle) with and without pre-nitridation treatment of substrate.

In the end of this letter, we calculated the GaN nanodots density on Si from their SEM images to investigate the density control by different growth parameters. Firstly, Figure 7 shows GaN nanodots density as function of the substrate temperature and their SEM images. The lower substrate temperature we set during the Ga droplet formation, the higher density of GaN nanodots we get. For the sample grown at higher substrate temperature, the surface diffusion rate of Ga ad-atoms is high. Ga ad-atoms re-evaporate and aggregate to form Ga droplet more easily, which makes the Ga droplets lower density. For lower density Ga droplets on Si, we get lower density GaN nanodots after plasma nitridation. Secondly, we can also find the pre-nitridation treatment of Si substrate make higher density of GaN nanodots from the result of the two samples at substrate temperature 500°C in the blue cycle of Figure 7. From the results of RHEED in section In situ RHEED analysis, pre-nitridation process forms an amorphous nitride layer on the surface which provides nitrogen atoms for Ga nucleating heterogeneously. Nitridized surface might reduce the diffusion length of Ga ad-atoms. Therefore, the sample with substrate pre-nitridation has higher density of GaN nanodots.

**Figure 8:**
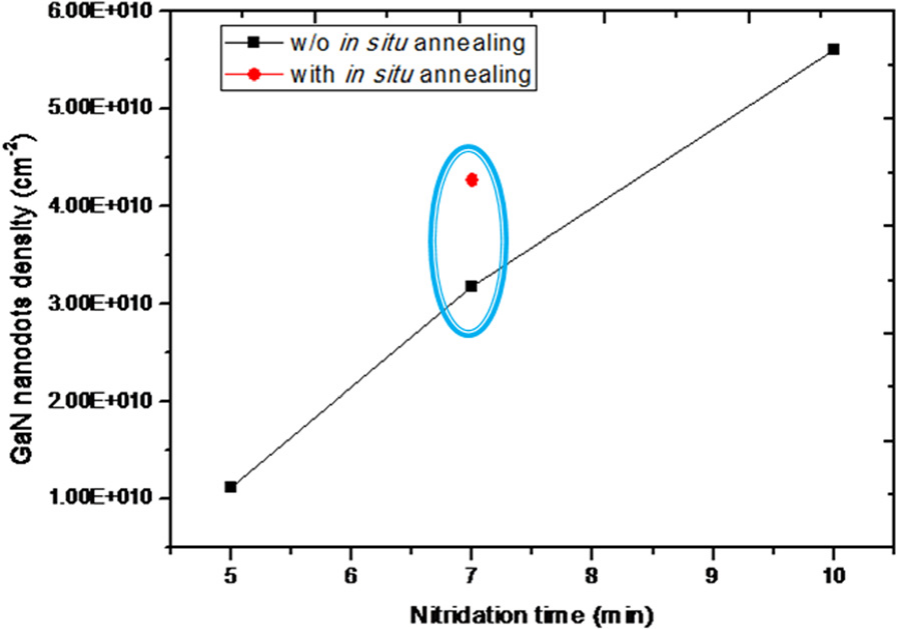
**Density of GaN nanodots as function of nitridation time for 5 min, 7 min and 10 min.** Density of GaN nanodots for two samples (in the blue cycle) with and without in situ annealing process.

Thirdly, we discuss GaN nanodots density as function of nitridation time as shown in Figure 8. The longer nitridation time we set, the higher GaN nanodots density we get. From the growth mechanism of GaN nanodots by droplet epitaxy, we know that one Ga droplet becomes several GaN nanodots during nitridation. Therefore, sufficient nitridation time provides more nitrogen source to form more GaN nanodots. Finally, *in situ* annealing process can also make the GaN nanodots density increase from the two samples of nitridation time 7 min shown in the blue cycle of Figure 8. As the result of XPS shows, *in situ* annealing not only makes higher degree of nitridation, but also forms higher density of GaN nanodots.

## Conclusions

In conclusion, we have demonstrated self-assembled and crystal GaN nanodots grown on Si (111) by droplet epitaxy using our PAMBE system. Their density can be controlled from 1.1 × 10^10^ to 1.1 × 10^11^ cm^-2^. The growth of GaN nanodots includes the Ga droplets nucleation, growth, coalescence, and GaN crystallization by plasma nitrogen source. *In situ* RHEED, FESEM, HRTEM, SPEM and XPS were employed to investigate the formation of GaN nanodots. Through the understanding of their growth mechanism, density of GaN nanodots can be controlled by the parameters: substrate temperature of Ga droplet formation, pre-nitridation of Si substrate, nitridation time for GaN crystallization, and *in situ* annealing after GaN nanodot formation.

## Abbreviations

LED: Light emitting diode; HEMT: High electron mobility transistor; GaN: Gallium nitride; QD: Quantum dot; MBE: Molecular beam epitaxy; MOCVD: Metal organic chemical vapor deposition; SPEM: Scanning photoemission microscopy; XPS: X-ray photoelectron spectroscopy; RHEED: Reflection high-energy electron diffraction; TEM: Transmission electron microscopy; FESEM: Field emission scanning electron microscopy.

## Competing interests

The authors declare that they have no competing interests.

## Authors' contributions

ISY carried out the design of the study and drafted the manuscript. CPC and CPY carried out all the experimental work. CTL participated in the design of the study. YRM performed the measurement of SPEM and XPS. CCC carried out the experiment of TEM. All authors read and approved the final manuscript.

## References

[B1] Nakamura S Pearton S Fasol G The Blue Laser Diode, The Complete Story 2000 2 Springer 3 5

[B2] Su M Chen C Rajan S Prospects for the application of GaN power devices in hybrid electric vehicle drive systems Semicond ScI Technol 2013 28 074012 10.1088/0268-1242/28/7/074012

[B3] Chow WW Schneider HC Theory of laser gain in InGaN quantum dots Appl Phys 2002 81 2566

[B4] Ji LW Su YK Chang SJ Liu SH Wang CK Tsai ST Fang TH Wu LW Xue QK InGaN quantum dot photodetectors Solid State Electron 2003 47 1753 10.1016/S0038-1101(03)00159-X

[B5] Fujii K Kato T Sato K Im I Chang J Yao T Photoelectrochemical application of GaN nanostructures on Si for hydrogen generation by water reduction Phys Stat Solidi C 2010 7 2218 2220 10.1002/pssc.200983449

[B6] Shen CH Lin HW Lee HM Wu CL Hsu JT Gwo S Self-assembled InN quantum dots grown on AlN/Si(111) and GaN/Al_2_O_3_(0001) by plasma-assisted molecular-beam epitaxy under stranski-krastanow mode thin solid films Thin Solid Films 2006 494 79 83 10.1016/j.tsf.2005.08.216

[B7] Brown J Wu F Petroff PM Speck JS GaN quantum dot density control by rf-plasma molecular beam epitaxy Appl Phys Lett 2004 84 690 692 10.1063/1.1645333

[B8] Mashanov VI Ulyanov VV Timofeev VA Nikiforov AI Pchelyakov OP Yu IS Cheng HH Formation of Ge-Sn nanodots on Si(100) surfaces by molecular beam epitaxy Nanoscale Res Lett 2011 6 85 10.1186/1556-276X-6-85 21711584 PMC3212234

[B9] Miyamura M Tachibana K Someya T Arakawa Y Stranski–Krastanow growth of GaN quantum dots by metalorganic chemical vapor deposition J Cryst Growth 2002 237–239 1316 1319

[B10] Zhang J Li S Xiong H Tian W Li Y Fang Y Wu Z Dai J Xu J Li X Chen C Fabrication of low-density GaN/AlN quantum dots via GaN thermal decomposition in MOCVD Nanoscale Res Lett 2014 9 341 10.1186/1556-276X-9-341 25136276 PMC4128446

[B11] Wu J Hirono Y Li X Wang ZM Lee J Benamara M Luo S Mazur Y Kim ES Salamo GJ Self-assembly of multiple stacked nanorings by vertically correlated droplet epitaxy Adv Funct Mater 2014 24 530 535 10.1002/adfm.201302032

[B12] Fuster D Gonzalez Y Gonzalez L Fundamental role of arsenic flux in nanohole formation by Ga droplet etching on GaAs(001) Nanoscale Res Lett 2014 9 309 10.1186/1556-276X-9-309 24994962 PMC4071335

[B13] Mano T Kuroda T Mitsuishi K Nakayama Y Noda T Sakoda K GaAs/AlGaAs quantum dot laser fabricated on GaAs (311)*A* substrate by droplet epitaxy Appl Phys Lett 2008 93 203110 10.1063/1.3026174

[B14] Cavigli L Bietti S Accanto N Minari S Abbarchi M Isella G Frigeri C High temperature single photon emitter monolithically intergrated on silicon Appl Phys Lett 2012 100 231112 10.1063/1.4726189

[B15] Wu J Shao D Dorogan VG Li AZ Li S DeCuir EA Jr Manasreh MO Wang ZM Mazur YI Salamo GJ Intersublevel infrared photodetector with strain-free GaAs quantum dot pairs grown by high temperature droplet epitaxy Nano Lett 2010 10 1512 1516 10.1021/nl100217k 20356102

[B16] Wang Y Ozcan AS Sanborn C Ludwig KF Bhattacharyya A Chandrasekran R Moustakas TD Zhou L Smith DJ Real-time x-ray studies of gallium nitride nanodot formation by droplet heteroepitaxy J Appl Phys 2007 102 073522 10.1063/1.2786578

[B17] Kondo T Saitoh K Yamamoto Y Maruyama T Naritsuka S Fabrication of GaN dot structures on Si substrates by droplet epitaxy Phys Sta Sol A 2006 203 1700 1703 10.1002/pssa.200565212

[B18] Wu CL Chou LJ Gwo S Size- and shape-controlled GaN nanocrystals grown on Si(111) substrate by reactive epitaxy Appl Phys Lett 2004 85 2071 2073 10.1063/1.1787947

[B19] Madras G McCoy BJ Temperature effects on the transition from nucleation and growth to Ostwald ripening Chem Eng Sci 2004 59 2753 2765 10.1016/j.ces.2004.03.022

[B20] Li C Zeng ZQ Fan DS Hirono Y Wu J Morgan TA Hu X Yu SQ Wang ZM Salamo GJ Bismuth nano-droplets for group-V based molecular-beam droplet epitaxy Appl Phys Lett 2011 99 243113 10.1063/1.3666036

[B21] Somaschini C Bietti S Trampert A Jahn U Hauswald C Riechert H Sanguinetti S Geelhaar L Control over the number density and diameter of GaAs nanowires on Si(111) mediated by droplet epitaxy Nano Lett 2013 13 3607 3613 10.1021/nl401404w 23898953

[B22] Debnath RK Stoica T Besmehn A Jeganathan K Sutter E Meijers R Luth H Calarco R Formation of GaN nandots on Si(111) by droplet nitridation J Cryst Growth 2009 311 3389 3394 10.1016/j.jcrysgro.2009.04.025

[B23] Chen CH Wang SC Yeh CM Hwang J Klauser R A scanning photoelectron microscopy study of AlN/SixNy insulating stripes Surf Sci 2005 599 107 10.1016/j.susc.2005.09.041

[B24] Family F Meakin P Kinetics of droplet growth-processes – simulations, theory, and experiments Phys Rev A 1989 40 3836 10.1103/PhysRevA.40.3836 9902602

[B25] Naritsukaa BS Kondoa T Otsuboa H Saitoha K Yamamotob Y Maruyama T In situ annealing of GaN dot structures grown by droplet epitaxy on (111) Si substrates J Cryst Growth 2007 300 118 122 10.1016/j.jcrysgro.2006.11.002

